# Supplementary material for: Identification and functional analysis of non-coding regulatory small RNA FenSr3 in Bacillus amyloliquefaciens LPB-18
Source: PeerJ. 2023 May 15;11:e15236. doi: 10.7717/peerj.15236 (PMC10194069; doi:10.7717/peerj.15236)
Supplement: Supplemental Information 4 [file peerj-11-15236-s004.zip › KO/CK-vs-T1_map/map05168.html]

KEGG PATHWAY: Herpes simplex infection - Reference pathway


|  |  |
| --- | --- |
| **Herpes simplex infection - Reference pathway** |  |

[
Pathway menu
| Organism menu
| Pathway entry
| Show description
| User data mapping
]

Herpes simplex virus (HSV) infections are very common worldwide, with the prevalence of HSV-1 reaching up to 80%-90%. Primary infection with HSV takes place in the mucosa, followed by the establishment of latent infection in neuronal ganglia. HSV is the main cause of herpes infections that lead to the formation of characteristic blistering lesion. HSV express multiple viral accessory proteins that interfere with host immune responses and are indispensable for viral replication. Among these proteins, the immediate early (IE) gene ICP0, ICP4, and ICP27 are essential for regulation of HSV gene expression in productive infection. On the other hand, ORF P and ORF O gene are transcribed during latency and blocks the expression of the IE genes, thus maintaining latent infection.

|  |  |  |
| --- | --- | --- |
| Reference pathway | 184% 150% 122% 100% 82% 67% 55% | 图片下载 |
